# Tauroursodeoxycholic acid enhances the development of porcine embryos derived from *in vitro*-matured oocytes and evaporatively dried spermatozoa

**DOI:** 10.1038/s41598-017-07185-w

**Published:** 2017-07-28

**Authors:** Xiao-Xia Li, Yun-Fei Diao, Hai-Jun Wei, Shi-Yong Wang, Xin-Yan Cao, Yu-Fei Zhang, Tong Chang, Dan-Li Li, Min Kyu Kim, Baozeng Xu

**Affiliations:** 1 Institute of Special Animal and Plant Sciences, Chinese Academy of Agricultural Sciences, Jilin, P. R. China; 2 State Key Laboratory for Molecular Biology of Special Economic Animals, Institute of Special Animal and Plant Sciences, Chinese Academy of Agricultural Sciences, Jilin, P. R. China; 3 Department of Animal Science and Biotechnology, College of Agriculture and Life Science, Chungnam National University, Daejeon, 305-764 Korea

## Abstract

Evaporative drying (ED) is an alternative technique for long-term preservation of mammalian sperm, which does not require liquid nitrogen or freeze-drying equipment, but offers advantages for storage and shipping at ambient temperature and low cost. However, the development of zygotes generated from these sperms was poor. Here, we demonstrated that the supplementation of tauroursodeoxycholic acid (TUDCA), an endogenous bile acid, during embryo culture improved the developmental competency of embryos derived from *in vitro* matured pig oocytes injected intracytoplasmically with boar ED spermatozoa by reducing the production of reactive oxygen species, the DNA degradation and fragmentation, and the expression of apoptosis-related gene Bax and Bak, and by increasing the transcription of anti-apoptosis gene Bcl-XL and Bcl-2. Furthermore, TUDCA treatment promoted the blastocyst quality manifested by the total cell numbers and the ratio of inner cell mass. Taken together, our data suggest that evaporative drying would be a potentially useful method for the routine preservation of boar sperm in combination with further optimization of subsequently embryo culture conditions.

## Introduction

Long-term preservation of mammalian sperm has widespread applications in medicine and agriculture, including facilitating treatment of human infertility and fertility preservation for male cancer patients, artificial insemination and thus genetic improvement of livestock, conservation of endangered species and maintenance of genetic diversity. While cryopreservation of mammalian sperm in liquid nitrogen (LN_2_) has been successfully employed, the ability to preserve sperm at much higher temperatures (ideally at ambient temperature) is preferred for a number of reasons. It would dramatically reduce the storage expenses, facilitate sample transport while reducing associated costs, and prevent cross contamination between samples stored in LN_2_^1–4^. Intracytoplasmic sperm injection (ICSI), an *in vitro* fertilization procedure in which a single sperm is injected directly into an egg, has truly revolutionized not only the treatment options for couples with impaired semen quality, and those with both obstructive and non-obstructive azoospermia, but also the methods of long-term sperm storage which just need to protect sperm DNA from physical damage irrespective of sperm motility or integrity of sperm membrane. Correspondingly, the new approaches, such as freeze-drying and evaporative-drying^5^, can long-term preserve mouse spermatozoa without using LN_2_ or dry ice for storage and transportation. Furthermore, the method of evaporative-drying also has an advantage over that of freeze-drying since the former does not need freeze-drying apparatus during sample preparation. However, whether the method of evaporative drying available for practical use in other animals, especially in swine, which serves as a well-recognized model for biomedicine research, needed further studies.

Preimplantation embryos are extremely sensitive to oxidative stress. For a rapid growth and differentiation, embryos need to produce more energy through electron transport chain in the mitochondria. With ATP production, reactive oxygen species (ROS) are also created as by-products in the mitochondria. In the normal physical condition, the production of ROS is sensitively controlled by the balance of oxidizing and reducing substances and these related enzymes. Accumulating evidence has shown that imbalance of oxidation and reduction leads to oxidative stress, which causes the developmental arrest, physical DNA damage, impaired expression profile of several anti- and pro-apoptotic members of the Bcl-2 family, apoptosis^6^, and embryo fragmentation^7, 8^. Thus, ROS homeostasis is one of the key factors to keep embryo healthy^9, 10^. On the other hand, tauroursodeoxycholic acid (TUDCA) is an endogenous bile acid synthesized in the liver conjugation pathway of ursodeoxycholic acid (UDCA), which is an FDA-approved molecule widely used in treatment of liver diseases and served as a potent inhibitor of apoptosis^11, 12^. Several reports have shown that TUDCA is an orally bioavailable and effective inhibitor of apoptosis by exhibiting antioxidant properties, maintaining mitochondrial membrane potential, and preventing cytochrome c release, Bax translocation, caspase activation during mitochondrial pathway of cell death as well as endoplasmic reticulum stress-mediated cell death in central nervous system and in liver^13–15^. Since vitrification of sperm has been shown to be closely associated with increased levels of reactive oxygen species (ROS) and apoptotic events^16–18^. It is tempting to speculate that addition of antioxidant TUDCA during embryo culture to reduce the levels of ROS in embryos derived from evaporative drying spermatozoa can improve the poor outcome following ICSI.

Therefore our objectives were to determine: (1) the feasibility that preservation of boar sperm through evaporative-drying technique; (2) the effects of supplementation of antioxidant TUDCA during embryo culture on the developmental competency of embryos derived from *in vitro* matured porcine oocyte injected with evaporative drying boar sperm. Our results demonstrate that TUDCA treatment increased the development and the quality of embryos from spermatozoa evaporatively dried in swine.

## Results

### Optimal TUDCA supplementation improved the preimplantation development following porcine cumulus-oocyte complexes (COCs) maturation *in vitro* and ICSI of evaporative drying boar sperms

To evaluate the feasibility that storage of boar sperm through evaporative-drying technique, we examined the development of embryos derived by ICSI of evaporatively dried spermatozoa following porcine COCs maturation *in vitro*. Compared to control embryos derived by ICSI of conventional (cryopreservation) spermatozoa following porcine COCs maturation *in vitro*, the cleavage rate (84.0 ± 1.9% *vs* 74.7 ± 2.2%; *p* < 0.05) and the percentages of blastocyst (23.3 ± 2.4% *vs* 15.5 ± 1.2%; *p* < 0.05) were significantly lower in the group of embryos generated using evaporatively dried spermatozoa (Table 1). To test whether reducing ROS by adding antioxidant TUDCA in the embryo culture medium increases the developmental competency of embryos derived from evaporative drying spermatozoa, we cultured embryos supplemented with 0, 100, 200 or 300 μM TUDCA, respectively. As shown in Table 1, 200 μM TUDCA could dramatically enhance the percentage of 2-cell, 4–8 cell, morula and blastocyst embryos when compared to other TUDCA-treated groups (0, 100, and 300 μM TUDCA, respectively) (N = 8, *p* < 0.05). Although embryos derived from evaporative drying spermatozoa in the 200 μM TUDCA-treatment group had less developmental competency as regards the rate of 2-cell embryo, morula and blastocyst than these of embryos generated using cryopreserved spermatozoa without TUDCA treatment (N = 8, *p* < 0.05), 200 μM TUDCA supplementation significantly increased the percentage of expanded and hatched blastocyst of embryos derived from evaporatively dried spermatozoa, compared to that of embryos generated using cryopreserved spermatozoa (N = 5, *p* < 0.05) (Table 2). In consistent with the rate of expanded and hatched blastocyst, the quality of embryos manifested by the total cell numbers and the ratio of inner cell mass was significantly higher in 200 μM TUDCA treatment group than that in without TUDCA treatment group and cryopreserved spermatozoa group (N = 3, *p* < 0.05) (Table 3). These results suggest that evaporative drying could be a routine method of porcine sperm preservation when in combination with 200 μM TUDCA supplementation during the subsequently embryo culture.

**Table 1:** Effect of different concentrations of TUDCA addition during embryo culture on the development of embryos.

Groups*	No. of oocytes	Cleavage (%, mean ± SD)	4–8 cell (%, mean ± SD)	>8 cell (%, mean ± SD)	Morula (%, mean ± SD)	Blastocyst (%, mean ± SD)
FT	182	84.0 ± 1.9^a^	75.3 ± 2.6^a^	67.2 ± 2.3^a^	50.8 ± 2.2^a^	23.3 ± 2.4^a^
ED+ 0 μM	184	74.7 ± 2.2^b^	62.2 ± 2.6^b^	52.4 ± 2.4^b^	36.5 ± 1.7^b^	15.5 ± 1.2^b^
ED+ 100 μM	184	75.8 ± 2.0^b^	64.3 ± 2.8^b^	54.3 ± 1.4^b^	37.1 ± 2.0^b^	16.0 ± 2.0^b^
ED+ 200 μM	185	81.3 ± 1.9^c^	74.1 ± 1.3^a^	65.5 ± 1.5^a^	47.6 ± 1.7^c^	19.6 ± 2.4^c^
ED+ 300 μM	185	78.9 ± 2.1^d^	68.3 ± 1.6^c^	59.5 ± 2.3^c^	37.5 ± 2.1^b^	16.1 ± 1.9^b^

Note: Different letters within the same column indicate statistical differences at *p* < 0.05 by one-way ANOVA followed by the LSD multiple comparison test (N = 8). FT: blastocysts developed from conventional freeze-thaw boar spermatozoa without TUDCA-treatment; ED+ 0 μM, ED+ 100 μM, ED+ 200 μM and ED+ 300 μM: blastocysts developed from evaporatively dried boar spermatozoa with 0, 100, 200 and 300 μM TUDCA-treatment, respectively.

**Table 2:** Effect of TUDCA supplementation during embryo culture on the production of expanded and hatched blastocyst.

Groups	No. of blastocyst	Expanded blastocyst (%, mean ± SD)	Hatched blastocyst (%, mean ± SD)
FT	50	65.9 ± 2.9^a^	25.9 ± 2.9^a^
ED	48	60.3 ± 3.1^b^	21.1 ± 2.6^b^
ED+ TUDCA	50	74.1 ± 4.6^c^	35.9 ± 4.3^c^

Note: Different letters within the same column indicate statistical differences at *p* < 0.05 by one-way ANOVA followed by the LSD multiple comparison test (N = 5). FT: blastocysts developed from conventional freeze-thaw boar spermatozoa without TUDCA-treatment; ED and ED+ TUDCA: blastocysts developed from evaporatively dried boar spermatozoa with 0 and 200 μM TUDCA-treatment, respectively.

**Table 3:** Effect of TUDCA supplementation during embryo culture on the quality of blastocysts.

Groups	No. of blastocyst	ICM (mean ± SD)	TE (mean ± SD)	Total (mean ± SD)	ICM/Total (%, mean ± SD)
FT	30	5.1 ± 0.1^a^	25.9 ± 0.4^a^	31.0 ± 0.4^a^	16.6 ± 0.4^a^
ED	30	3.6 ± 0.1^b^	21.4 ± 0.4^b^	25.0 ± 0.4^b^	14.4 ± 0.5^b^
ED+ TUDCA	30	7.0 ± 0.2^c^	27.5 ± 0.1^c^	34.5 ± 0.3^c^	20.3 ± 0.3^c^

Note: Different letters within the same column indicate statistical differences at *p* < 0.05 by one-way ANOVA followed by the LSD multiple comparison test (N = 3). FT: blastocysts developed from conventional freeze-thaw boar spermatozoa without TUDCA-treatment; ED and ED+ TUDCA: blastocysts developed from evaporatively dried boar spermatozoa with 0 and 200 μM TUDCA-treatment, respectively. ICM: cell numbers of the inner cell mass; TE: numbers of trophoblast cells; Total: cell numbers of the inner cell mass plus numbers of trophoblast cells.

### TUDCA supplementation enhanced the developmental competency of porcine embryos derived from evaporative drying boar sperms by decreasing the levels of ROS and apoptosis

**Figure 1:**
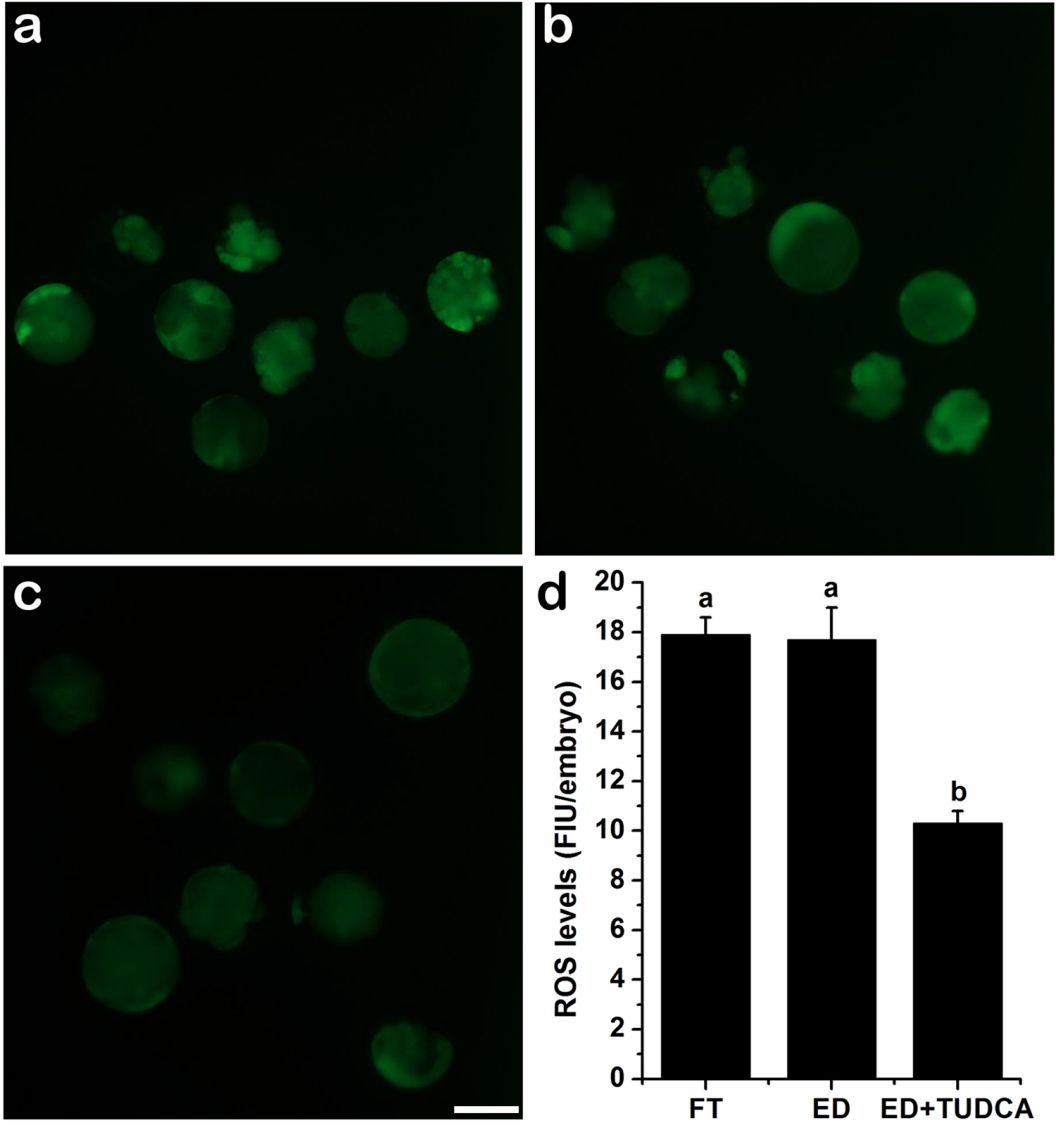
Effect of TUDCA supplementation during embryo culture on the levels of intracellular reactive oxygen species (ROS) in porcine blastocysts. Representative fluorescence images of blastocysts from *in vitro* matured oocytes fertilized using ICSI with the conventional freeze-thaw (FT) boar spermatozoa without TUDCA-treatment (**a**), evaporatively dried boar spermatozoa without TUDCA-treatment (**b**) or evaporatively dried boar spermatozoa with 200 μM TUDCA-treatment (**c**). Bar: 100 μm. d. ROS contents were quantified as fluorescent intensity for three independent times. Each time 6–12 blastocysts were measured. Each bar represents the mean ± SD of the fluorescent density. Bars that do not share the same letter are significantly different at *p* < 0.05.

**Figure 2:**
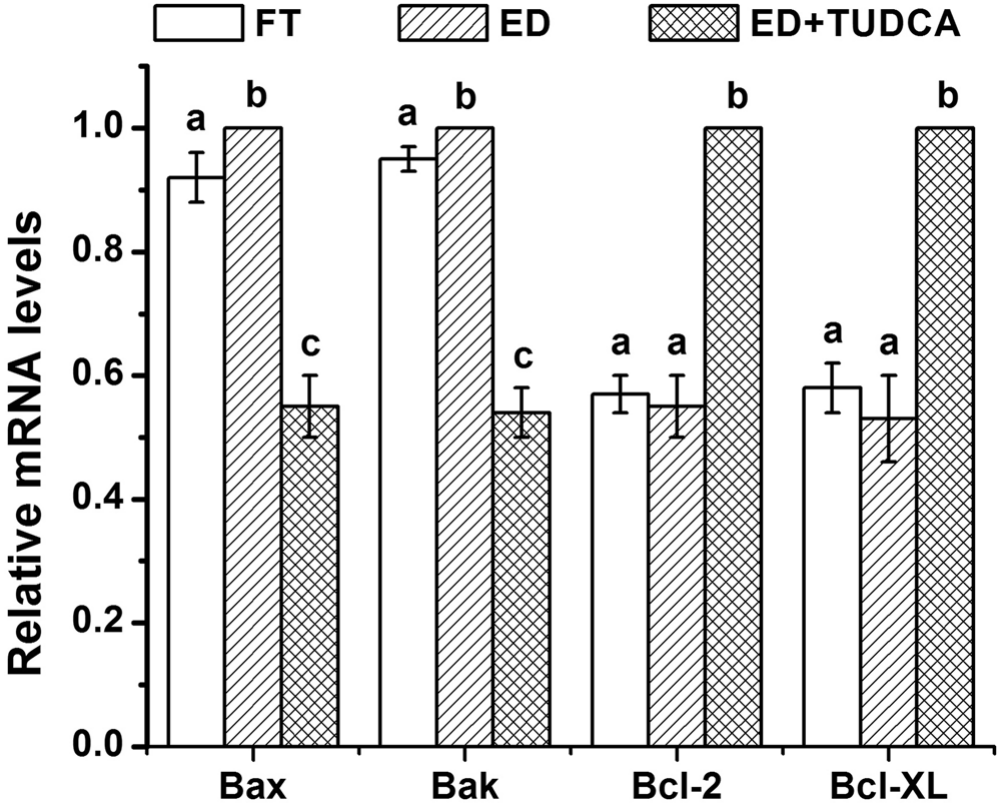
Relative transcript levels of pro-apoptotic gene of Bax and Bak and anti-apoptotic gene of Bcl-XL and Bcl-2 in blastocysts with or without TUDCA treatment. FT: Blastocysts developed from conventional freeze-thaw boar spermatozoa without TUDCA-treatment; ED: Blastocysts developed from evaporatively dried boar spermatozoa without TUDCA-treatment; ED+ TUDCA: Blastocysts developed from evaporatively dried boar spermatozoa with 200 μM TUDCA-treatment. mRNA levels of Bax and Bak in FT and ED+ TUDCA groups were normalized against those in ED group. GAPDH served as a loading control. Each bar represents the mean ± SD. All experiments were repeated for three times. Bars that do not share the same letter are significantly different at *p* < 0.05.

**Figure 3:**
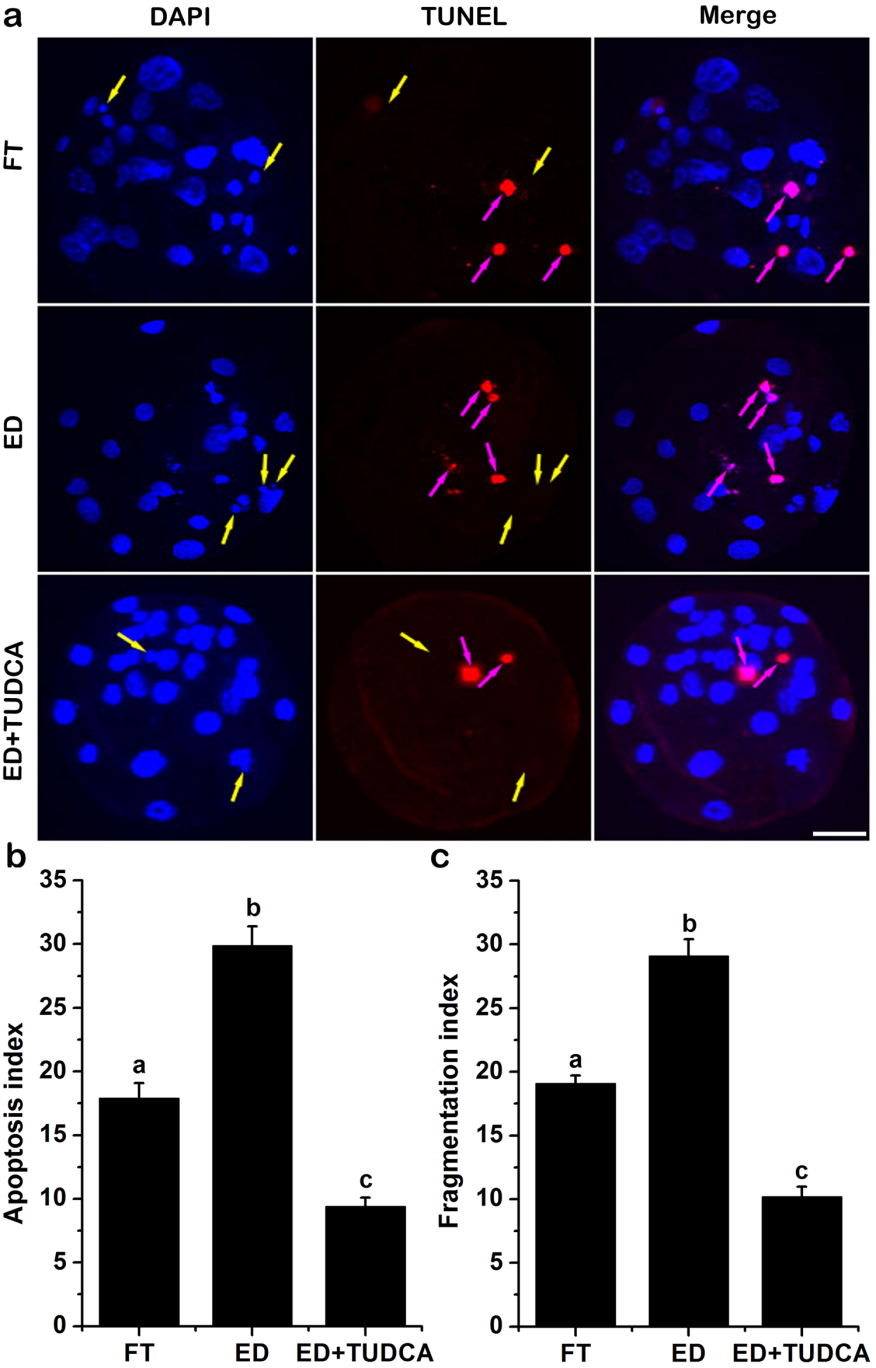
Effects of TUDCA addition during embryo culture on the quality of porcine blastocysts. (**a**) Representative fluorescence images of TUNEL staining. Blue: DNA; Red: apoptotic cell; Bar: 30 μm. The indices of apoptosis (**b**) and fragmentation (**c**) are expressed as mean ± SD for four times. Different low case letters above columns indicate statistical differences at *p* < 0.05 by one way ANOVA followed by LSD test. FT: Blastocysts developed from conventional freeze-thaw boar spermatozoa without TUDCA-treatment; ED: Blastocysts developed from evaporatively dried boar spermatozoa without TUDCA-treatment; ED+ TUDCA: Blastocysts developed from evaporatively dried boar spermatozoa with 200 μM TUDCA-treatment.

To assess how does TUDCA increase the embryo development and quality, we first compared the levels of ROS in embryos generated using evaporatively dried spermatozoa with or without 200 μM TUDCA treatment and embryos derived from cryopreserved spermatozoa. As shown in Fig. 1d, the levels of ROS were comparable between embryos generated using evaporatively dried spermatozoa without TUDCA treatment (17.7 ± 1.3, n = 31) and embryos derived from cryopreserved spermatozoa (17.9 ± 0.7, n = 36). While 200 μM TUDCA treatment significantly decreased the ROS levels in the embryos generated using evaporatively dried spermatozoa (10.3 ± 0.5%, n = 33) compared to those of using either cryopreserved spermatozoa or evaporative drying spermatozoa without TUDCA treatment (N = 3, *p* < 0.05). Since high level of ROS has been shown to be closely associated with apoptotic events, we then tested the transcript levels of genes involved in apoptosis. Our results demonstrated that the expression of proapoptosis genes Bax and Bak was significantly lower in embryos generated using cryopreserved spermatozoa group than that in embryos derived from evaporatively dried spermatozoa without TUDCA treatment. As expected, 200 μM TUDCA dramatically reduced the expression of Bax and Bak in embryos derived from evaporatively dried spermatozoa to a level that was even lower than that in embryos generated using cryopreserved spermatozoa (N = 3, *p* < 0.05). On the other hand, the expression levels of anti-apoptosis genes Bcl-XL and Bcl-2 were comparable between embryos derived from evaporatively dried spermatozoa without TUDCA treatment and embryos generated using cryopreserved spermatozoa. More interestingly, 200 μM TUDCA dramatically increased the expression of Bcl-XL and Bcl-2 in the embryo generated using evaporatively dried spermatozoa (N = 3, *p* < 0.05) (Fig. 2). Finally, we detected the effect of TUDCA on the occurrence of apoptosis in the embryos derived from evaporatively dried spermatozoa with TUNEL assay and embryo fragmentation. As shown in Fig. 3, both TUNEL (17.9 ± 1.2%, n = 32 *vs* 29.9 ± 1.5%, n = 32) and fragmentation (19.1 ± 0.6%, n = 32 *vs* 29.1 ± 1.3%, n = 32) index were significantly lower in embryos generated using cryopreserved spermatozoa than that in embryos derived from evaporatively dried spermatozoa without TUDCA treatment. Consistently, 200 μM TUDCA dramatically cut down both TUNEL (9.4 ± 0.7%, n = 32) and fragmentation (10.2 ± 0.8%, n = 32) index in embryos derived from evaporatively dried spermatozoa to a level that was much lower than that in embryos generated using cryopreserved spermatozoa (N = 4, *p* < 0.05).

## Discussion

Our current results demonstrate that zygotes derived by ICSI of evaporatively dried boar spermatozoa following porcine COCs maturation *in vitro* developed to blastocyst less frequently than controls derived by ICSI of conventional (cryopreservation) spermatozoa. We predicted that the sperm damage caused by evaporative drying interferes with the physical DNA damage and embryo fragmentation, which makes the subsequent embryos vulnerable to oxidative stress. In fact, we found that the supplementation of antioxidant TUDCA during embryo culture enhanced the developmental competency of embryos derived from evaporative drying spermatozoa in a dose-dependent manner through decreasing the production of ROS and the occurrence of apoptosis. Our results suggest that the preservation of boar sperm through evaporative-drying technique is feasible when combination with further optimization of subsequently embryo culture conditions.

Because of its ease of application and cost-effectiveness, evaporative drying has been considered an attractive alternative to traditional methods for preserving wild and genetically modified mouse spermatozoa on a variety of genetic backgrounds, including B3C3F1, C57BL/6, and FVB/N^19–23^. To date, however, there have been few studies to validate the use of evaporative drying technology to preserve, store, and recover boar spermatozoa. Earlier studies have shown that residual centrosomes of spermatozoa play different roles for the reconstruction of the zygotic centrosome after fertilization between non-rodent and rodent mammals. In rodents, sperm centrioles degenerate during spermatogenesis, and the sperm brings little or no centrosome material into the egg at fertilization^24, 25^. Whereas in pigs and other non-rodent mammals, sperm centrioles are not completely lost, and so the sperm delivers one intact and one partially degenerate centriole to the egg at fertilization^26, 27^. Thus, porcine ova cannot be fertilized as soon as the evaporatively dried boar sperm is injected unless, beside a 70 kDa protein (phospholipase C zeta) which is necessary to activate mammalian eggs^28^, the activity of centrosomes of boar spermatozoa is not destroyed by evaporative drying.

It is well-known that *in vitro* embryonic development could be disturbed by high oxygen concentration, which imposes deteriorative microenvironment on preimplantation embryos through increasing the production of ROS^7, 29–33^. Moreover, some researchers showed that species-specific blocks to embryonic development could be induced by the rise of hydrogen peroxide levels attributable to *in vitro* culture^34, 35^. This effect of oxidative stress can be weakened by efficient antioxidant agents, such as catalase or superoxide dismutase, as well as thiol compounds acting as metabolic buffers which scavenge active oxygen species in most cells^36^. Castro *et al*.^37^ reported that TUDCA can directly inhibit the production of reactive oxygen species, eliminate the transmembrane potential and keep the outer mitochondrial membrane intact. Consistently, our data demonstrated that antioxidant TUDCA can lower the contents of reactive oxygen species (ROS), decrease the DNA degradation and fragmentation as well as apoptosis through optimizing the expression of apoptosis-related genes Bax and Bak, and anti-apoptosis genes Bcl-XL and Bcl-2 in porcine embryo *in vitro*. As a result, the quality of blastocyst was improved indicated as total cell number and the percentage of inner cell mass. On the other hand, ROS function as essential signal transducers regulating the normal cellular physiology, including the cellular gene expression pattern, the developmental breakdown of cellular organelles during differentiation or cell maturation and biotransformation of xenobiotics^38^. Our data also indicated that supplementation of extremely high concentration of TUDCA, like 300 μM, during embryo culture posed a potential hazard in the preimplantation developmental competency in swine manifested by a lower rate of cleavage and blastocyst embryo when compared to 200 μM TUDCA supplementation. Thus, our data suggest that optimal addition of antioxidant reagents during embryo culture could be beneficial for the development of mammalian embryos derived from evaporatively dried spermatozoa. In addition, apoptosis is regarded as a side effect of *in vitro* culture of embryos in bovine^39^ and porcine^40^. However, porcine blastocysts produced *in vivo* have little or no apoptotic DNA fragmentation^41^. Therefore, the degree of apoptosis in blastocysts can be used as an indicator for the effectiveness and suitability of the culture condition for embryo development^42^.

## Materials and Methods

All chemicals and reagents used in the current study were purchased from Sigma-Aldrich Chemical Company (St. Louis, MO,USA) unless otherwise indicated.

### Oocyte collection and maturation *in vitro*

Porcine ovaries were obtained from prepubertal gilts at a local slaughterhouse and transported to the laboratory within 2 h. Fully-grown GV-stage oocytes surrounded by cumulus cells, named Cumulus-oocyte complexes (COCs), were isolated from follicles (3–7 mm in diameter) with an 18-gauge needle fixed to a 10 ml disposable syringe. After washing 6 times with maturation medium I (MI-medium), which consisted of TCM-199 supplemented with 10% porcine follicular fluid (PFF), 10 ng/ml epidermal growth factor (EGF), 10 IU/ml Pregnant Mare Serum Gonadotropin (PMSG) and 10 IU/ml human Chorionic Gonadotrophin (hCG), oocytes with compact cumulus and evenly granulated ooplasm were collected for maturation *in vitro*. Each 50 COCs was firstly cultured in 500 μl maturation MI medium in a well of four-well multi-dish (Nunc, Roskilde, Denmark) at 38.5 °C in an atmosphere of 5% CO_2_ and saturated humidity for 22 h. Then COCs were transferred into MII-medium (PMSG and hCG were omitted from MI-medium) for the second 22 h of culture at the same conditions as the first 22 h.

### Semen collection, evaporative drying and rehydration of evaporatively dried boar spermatozoa

All animal experiments were conducted exactly in accordance with the guide to the Care and Use of Experimental Animal issued by the Animal Ethics Committee of Institute of Special Animal and Plant Sciences, Chinese Academy of Agricultural Sciences. In addition, these experimental procedures had been approved by the committee before we did our experiments. As previously mentioned in our study^43^, diluted semen (25 × 10^6^ sperm/ml) came from Jilin academy of agricultural sciences in China. Semen was collected from proved fertile adult Duroc boars at 15–22 months of age. The sperm-rich fractions of ejaculates with greater than 85% motile spermatozoa were used. After being transported to the laboratory within 1 h, the semen was centrifuged at 700 g for 5 min and the supernatant was removed by careful aspiration. Before the pellet was diluted with desiccation preservation solution (Tyrode based desiccation [TrB] buffer consisting of 5.69 g/l NaCl, 0.23 g/l KCl, 0.29 g/l CaCl_2_·2H_2_O, 0.08 g/l MgCl_2_·6H_2_O, 2.09 g/l NaHCO_3_, 0.04 g/l NaH_2_PO_4_, 0.9 g/l Glucose and 2.3 g/l HEPES) supplemented 0.2 M trehalose, the pellet was washed twice with Phosphate Buffered Saline (PBS) and TrB buffer, respectively^44^. The sperm pellet was re-suspended in TrB buffer at a concentration of 1 × 10^7^ sperm/ml. Following each 20 μl of sperm was placed on glass slide in the clean bench for 30 min, these evaporative-drying sperms were packed with a tinfoil and stored at 4 °C. For rehydration of evaporatively dried spermatozoa, sperms were submerged with the same volume (20 μl) of PBS containing 0.1% w/v polyvinyl alcohol (DPBS-PVA) for 5 min. And only normal shaped spermatozoa (with an intact head, long and straight tail) were used for ICSI. All the evaporative-drying experiments were performed at room temperature (about 24 °C).

### Conventional cryopreservation of spermatozoa

Sperms were washed three times with PBS by centrifugation at 700 g for 5 min each time. Then sperms were suspended by Tris buffer (pH7.4) supplement with 20% (vol/vol) egg yolk and kept at 4 °C for 1 h. After centrifugation, sperms were diluted with Tris buffer (pH7.4) supplement with 20% (vol/vol) egg yolk and 12% (vol/vol) glycerol to 1 × 10^6^ sperm/ml, and equilibrated at 4 °C for another 1 h. Finally, the sperm suspension was loaded into 0.25 ml straws. These straws were placed horizontally on a sieve, which was 4 cm above the surface of liquid nitrogen, for 10 min and then plunged into liquid nitrogen. For thawing, a straw of frozen sperms was rapidly transferred from liquid nitrogen into a water bath at 37.5 °C for 1 min, and then washed twice in 1.5 ml EP tube with 1 ml Dulbecco’s PBS supplemented with 0.1% w/v polyvinyl alcohol and 10% v/v FBS (DPBS-PVA + FBS). At last, sperms were suspended in 1 ml DPBS-PVA + FBS buffer waiting for intracytoplasmic sperm injection.

### Intracytoplasmic sperm injection (ICSI)

As previously mentioned in our studies^45, 46^, ICSI was carried out with a micromanipulator under an inverted microscope. Evaporative-drying sperm or immobilized spermatozoon by tail-scoring/cutting was sucked into the injection pipette. Then a holding pipette held the denuded metaphase II oocyte with uniform cytoplasm, whose polar body at either the 6 or 12 o’clock position. At the 3 o’clock position of the oocyte, injection pipette pierced through the oocyte. Small amount of cytoplasm was aspirated to break the plasma membrane when the tip of the injection pipette had been inserted approximately on third of the way to half way through the oocyte. The aspirated cytoplasm with a spermatozoon was then gently expelled into the oocyte. All the procedures were performed at room temperature (about 24 °C).

### Embryo culture and assessment of embryonic development

Each 10 embryos derived from oocytes after ICSI of either freeze-thaw (FT) boar spermatozoa or evaporatively dried boar spermatozoa was cultured in a 40 μl drop of PZM-3 medium supplemented with 0 μM, 100 μM, 200 μM or 300 μM TUDCA as experiment required. All medium drops were covered by mineral oil and incubated at 38.5 °C in a humidified air of 5% CO_2_. The rates of cleavage, 4- to 8-cell embryo, morula and blastocyst embryo were determined at 24, 48–72, 144 and 168 h after embryo culture, respectively.

### Dual Differential Staining

The ICM and trophectoderm cells of blastocysts were evaluated with differential staining using a previously described technique^47^. Briefly, blastocysts were firstly removed the zona pellucida with 0.5% (w/v) pronase solution. Then the zona pellucida-free blastocysts were exposed to a 1:5 dilution of rabbit anti-pig whole serum for 1 h. After washing in TL-HEPES medium containing 1 mg/ml of PVA, the blastocysts were transferred to a solution consisting of a 1:10 dilution of guinea pig complement containing 10 μg/ml of propidium iodide (PI) and 10 μg/ml of bisbenzimide (Hoechst 33342) for 1 h. The blastocysts were then mounted on slides under cover slips and the cell counts were made directly under fluorescence microscope (TE2000-U, Nikon, Japan). The nuclei of the ICM cells were labeled with bisbenzimide and appeared blue, while the nuclei of the TE cells were stained with both fluorochromes and thus were fluorescing pink.

### Reverse transcription polymerase chain reaction (RT-PCR)

Total RNA was isolated from a pool of 50 blastocysts by using Trizol reagent (Gibco BRL, Cat. No. 15596) according to the manufacturer’s instruction. First-strand cDNA synthesis was carried out using cDNA synthesis kit (Bio-Rad, Hercules, CA) as described previously^45^. Each cDNA sample was processed for PCR amplification by denaturation at 95 °C for 5 min, followed by 35 to 45 cycles at 95 °C for 30 s, 57 °C for 30 s, and 72 °C for 30 s, and final extension at 72 °C for 10 min. The primers used for RT-PCR are given in Table 4. After PCR amplifications, the PCR products were applied to 2% agarose gel electrophoresis in TAE buffer and visualized with ethidium bromide staining. The intensity of each band was quantified and the relative intensity against GAPDH in the same lane was calculated by using ImageQuant (GE Healthcare).

**Table 4:** Primers used for RT-PCR analyses.

Gene	Primers (5′-3′)	Product size (base pair)	RT-PCR condition
BAX	F: AAGCGCATTGGAGATGAACT R: GGCCTTGAGCACCAGTTTAC	184	40 cycles/57 °C
BAK	F: CTGCCCCTAGAACCTAGCAG R: TTGATGCCACTCTCGAACAG	186	40 cycles/57 °C
BCL-2	F: ATGTGTGTGGAGAGCGTCAA R: CCTTCAGAGACAGCCAGGAG	188	45 cycles/57 °C
BCL-XL	F: ACAGCGTATCAGAGCTTTGAGCA R: CGTCAGGAACCATCGGTTGAAG	297	35 cycles/58 °C
GAPDH	F: TTCCACGGCACAGTCAAGGC R: CATGGTCGTGAAGACACCAG	151	40 cycles/57 °C

Note: F: forward primer; R: reverse primer.

### Assays for Intracellular ROS Levels in Embryos

The intracellular ROS levels of blastocyst were evaluated with Image-iT Live Green Reactive Oxygen Species Detection Kit (Invitrogen, Eugene, Oregon) according to the manufacturer’s instructions. Briefly, Day 6 blastocysts were incubated with tert-butyl hydroperoxide (TBHP) for 1 h to induce intracellular ROS production. The blastocysts were then stained with 5-(and-6)-carboxy-2′, 7′dichlorodihydrofluoresceindiacetate (carboxy-H2DCFDA) for 30 min and analyzed the intracellular ROS level under fluorescence microscope (TE2000-U, Nikon, Japan). ImageJ software 1.43 u (National Institutes of Health, USA) was used for quantitative analysis of fluorescent intensity of each embryo. Background taken just outside the cells was subtracted from each image.

### TUNEL assay

Apoptotic cells in the blastocysts were detected by using *In Situ* Cell Death Detection Kit (Roche Diagnostics, Indianapolis, IN). Day 7 blastocysts were fixed with 4% paraformaldehyde in PBS (pH 7.4) for 1 h at room temperature. Subsequently, the fixed blastocysts were permeabilized with 1.0% Triton X-100 and 0.1% sodium citrate in PBS for 0.5 h followed three times wash in PBS containing 0.1% PVA at room temperature. Then blastocysts were incubated with fluorescein-conjugated dUTP and terminal deoxynucleotidyl transferase according to the manufactuer’s instruction in the dark for 1 h at 37 °C. After thorough rinse, embryos were counterstained with DAPI (4′,6-diamidino-2-phenylindole) and mounted in the mounting medium (Vectashield, Vector Laboratories, Inc., Burlingame, CA). The number of total and apoptotic cells in each embryo were counted using scanning laser confocal microscope (LSM5 Live, Carl Zeiss, Jena, Germany). Apoptotic cells presenting pink color, which were double stained with TUNEL (red signal) and DAPI (blue signal); non-apoptotic cells and fragmented nuclear were labeled with DAPI and appeared blue. The apoptotic and fragmentation index was calculated for each embryo as follows: apoptotic index = (number of TUNEL-positive nuclei/total number of nuclei in blastocyst) × 100; fragmentation index = (number of fragmented cell/total number of cell in blastocyst) × 100.

### Statistical analysis

Results were analyzed by using SPSS 17.0 software. For each experiment, at least 30 embryos were measured. The data were analyzed by one way ANOVA followed by LSD. Data are expressed as mean values ± standard deviation. *P* < *0.05* was considered as significant difference.
